# Human Embryonic Stem Cell-Derived Neural Lineages as* In Vitro* Models for Screening the Neuroprotective Properties of* Lignosus rhinocerus* (Cooke) Ryvarden

**DOI:** 10.1155/2019/3126376

**Published:** 2019-08-19

**Authors:** Yin Yeo, Joash Ban Lee Tan, Lee Wei Lim, Kuan Onn Tan, Boon Chin Heng, Wei Ling Lim

**Affiliations:** ^1^Department of Biological Sciences, School of Science and Technology, Sunway University, 47500 Subang Jaya, Malaysia; ^2^School of Science, Monash University Malaysia, 47500 Subang Jaya, Malaysia; ^3^Li Ka Shing Faculty of Medicine, School of Biomedical Sciences, The University of Hong Kong, Pokfulam, Hong Kong; ^4^Peking University School of Stomatology, 100081 Haidian District, Beijing, China; ^5^Dean's office, Faculty of Science and Technology, Sunway University, 47500 Subang Jaya, Malaysia

## Abstract

In the biomedical field, there is growing interest in using human stem cell-derived neurons as* in vitro *models for pharmacological and toxicological screening of bioactive compounds extracted from natural products.* Lignosus rhinocerus *(Tiger Milk Mushroom) is used by indigenous communities in Malaysia as a traditional medicine to treat various diseases. The sclerotium of* L. rhinocerus *has been reported to have medicinal properties, including various bioactivities such as neuritogenic, anti-inflammatory, and anticancer effects. This study aims to investigate the neuroprotective activities of* L. rhinocerus *sclerotial extracts. Human embryonic stem cell (hESC)-derived neural lineages exposed to the synthetic glucocorticoid, dexamethasone (DEX), were used as the* in vitro *models. Excess glucocorticoids have been shown to adversely affect fetal brain development and impair differentiation of neural progenitor cells. Screening of different* L. rhinocerus *sclerotial extracts and DEX on the hESC-derived neural lineages was conducted using cell viability and neurite outgrowth assays. The neuroprotective effects of* L. rhinocerus *sclerotial extracts against DEX were further evaluated using apoptosis assays and Western blot analysis. Hot aqueous and methanol extracts of* L. rhinocerus *sclerotium promoted neurite outgrowth of hESC-derived neural stem cells (NSCs) with negligible cytotoxicity. Treatment with DEX decreased viability of NSCs by inducing apoptosis. Coincubation of* L. rhinocerus *methanol extract with DEX attenuated the DEX-induced apoptosis and reduction in phospho-Akt (pAkt) level in NSCs. These results suggest the involvement of Akt signaling in the neuroprotection of* L. rhinocerus *methanol extract against DEX-induced apoptosis in NSCs. Methanol extract of* L. rhinocerus *sclerotium exhibited potential neuroprotective activities against DEX-induced toxicity in hESC-derived NSCs. This study thus validates the use of human stem cell-derived neural lineages as potential* in vitro *models for screening of natural products with neuroprotective properties.

## 1. Introduction

Human embryonic stem cells (hESCs) derived from the inner cell mass of embryo at the blastocyst stage are pluripotent and have wide applications in tissue engineering and regenerative medicine [1, 2]. Neural differentiation of hESCs into neuronal cells can be achieved using chemically defined culture medium supplemented with small molecules [3]. Neural stem cells (NSCs) derived from hESCs can be further differentiated to produce the three major neural lineages: astrocytes, oligodendrocytes, and neurons and can serve as better* in vitro* models that recapitulate human neural physiology more accurately than animal-based models [2–4]. Additionally,* in vitro* models derived from hESCs may circumvent interspecies differences and thus reduce the uncertainty arising from the extrapolation of experimental data from animal to human models [4, 5]. Therefore, hESC-derived neural lineages can potentially serve as* in vitro* models for the modelling of neurodevelopmental and neurological disorders as well as for screening and identification of neuroactive and neurotoxic compounds [1, 3, 6].

*Lignosus rhinocerus*, also known as Tiger Milk Mushroom or “cendawan susu harimau” in the Malay language, is one of the most highly sought-after medicinal mushrooms by native communities in Malaysia [7, 8]. Its underground dense mass of hardened mycelia, known as the sclerotium, has much medicinal value [9]. Indigenous communities in Malaysia use the sclerotium of* L. rhinocerus* to cure various diseases, such as cough, asthma, lung and respiratory disease, fever, food poisoning, cancer, and wound healing [7, 10, 11]. It has been reported that this mushroom can also be used to decrease swelling in the body and act as a general tonic to enhance the overall well-being [10]. Indeed,* L. rhinocerus* is described as a national treasure of Malaysia because of its diverse medicinal properties [12]. Furthermore, the bioactivities of* L. rhinocerus *sclerotium have been reported in many* in vitro *and* in vivo *studies, including its antiasthmatic, anti-inflammatory, antimicrobial, antioxidant, and anticancer properties [13]. In particular, studies showed that* L. rhinocerus *sclerotial extracts could enhance neurite outgrowth in animal-derived* in vitro* models, which implies that this mushroom could potentially have effects on neuroregeneration [14, 15]. This also suggests the potential use of* L. rhinocerus* as a neuroprotective agent against neurotoxic drugs due to the presence of various neuroactive compounds. However, the* in vitro *neuritogenic and neuroprotective effects of* L. rhinocerus* sclerotium on human-derived neural lineages have not yet been demonstrated.

Glucocorticoids (GCs) are steroid hormones secreted mainly by the adrenal glands, which are involved in regulating responses to stress and intrauterine programming [16, 17]. Synthetic GCs, such as dexamethasone (DEX), are frequently used to treat severe complications linked to premature birth, thus lowering early neonatal mortality [18]. Moreover, DEX can also be used to promote lung maturation and to help avoid respiratory disorders in premature babies [19]. However, treatment using synthetic GCs has been reported to impair developing brain motor capacity and cognitive skills as well as increase the risk of cerebral palsy [18, 20]. Prenatal exposure to DEX has been reported to cause various detrimental effects such as decreased birth weight, elevated risk of cardiometabolic illness in children, and mood disorders in later life [17]. Many* in vivo* and* in vitro* studies have described the adverse effects of DEX, including reduced survival, decreased proliferation, and inhibited neurite outgrowth in animal-derived embryonic and adult neuronal cells [16, 20–23]. Moreover, it would be better to perform screening of potentially neuroprotective compounds against DEX in human-derived neuronal cells. Although various small molecules such as folic acid and melatonin have been reported to exhibit neuroprotection against DEX [24, 25], it would be of great interest to identify potential bioactive compounds from natural products that can confer protection to the nervous system. In this study, we aimed to investigate the potential neuroprotective activities of different* L. rhinocerus *sclerotial extracts against DEX-induced effects by using hESC-derived neural lineages as the* in vitro* models.

## 2. Materials and Methods

### 2.1. Chemicals, Culture Media, and Consumables

ROCK inhibitor Y27632, dimethyl sulfoxide (DMSO), poly-L-ornithine, N6,2'-O-Dibutyryladenosine 3',5'-cyclic monophosphate (dibutyryl cAMP), dexamethasone (DEX), Dulbecco's Modified Eagle's Medium (DMEM), all-trans-retinoic acid (RA), and phosphate buffered saline (PBS) were obtained from Sigma-Aldrich Inc. (St. Louis, MO, USA). Geltrex, Neurobasal medium, Neural Induction Supplement, Advanced DMEM/F-12, StemPro Accutase Cell Dissociation Reagent, KnockOut DMEM/F-12, GlutaMAX-I Supplement, basic fibroblast growth factor (bFGF), epidermal growth factor (EGF), StemPro Neural Supplement, B-27 Serum-Free Supplement, laminin, Penicillin-Streptomycin antibiotic solution, 0.25% trypsin-EDTA solution, collagenase, and 5'-bromo-2-deoxy-uridine (BrdU) were obtained from Gibco, Thermo Fisher Scientific Inc. (Waltham, MA, USA). StemMACS iPS-Brew XF medium was obtained from Miltenyi Biotec Inc. (Bergisch Gladbach, Germany). Fetal bovine serum (FBS) was purchased from Capricorn Scientific GmbH (Ebsdorfergrund, Germany). Cell culture consumables such as cell culture plates and flasks, cell culture chamber slides, and serological pipettes were obtained from SPL Life Sciences Co. (Korea).

### 2.2. Preparation of Aqueous and Methanol Extracts of* L. rhinocerus *Sclerotium

Sclerotium powder of* L. rhinocerus* (cultivar TM02) was obtained from Ligno Biotech Sdn. Bhd. (Selangor, Malaysia). For hot aqueous (HA) extraction, the* L. rhinocerus* sclerotium powder was soaked in distilled water (1:10, w/v) and double boiled for 30 min. The mixture was then cooled to room temperature and centrifuged at 4000 rpm for 15 min. For cold aqueous (CA) and room temperature aqueous (RT) extractions, the* L. rhinocerus* sclerotium powder was soaked in distilled water (1:10, w/v) and the mixture was stirred continuously for 1 h at 4°C and room temperature, respectively. For methanol (ME) extraction, the* L. rhinocerus* sclerotium powder was soaked in 80% (v/v) methanol (in distilled water) at a ratio of 1:10 (w/v) and stirred continuously at room temperature for 1 h. All mixtures were centrifuged at 4000 rpm for 15 min and the supernatant was filtered, and residues were then reextracted twice. The resulting* L. rhinocerus* aqueous extracts were freeze-dried and kept at −20°C prior to use, whereas the* L. rhinocerus* methanol extracts were evaporated using a rotary evaporator at 37°C. For downstream biochemical assays and neuroprotective studies, the* L. rhinocerus* aqueous extracts were redissolved in water and the* L. rhinocerus* methanol extracts were redissolved in 10% (v/v) DMSO.

### 2.3. Chemical Compositions of* L. rhinocerus *Sclerotial Extracts

The chemical compositions of aqueous and methanol* L. rhinocerus *sclerotial extracts were characterized by measuring the total carbohydrate, protein, and phenolic contents of the different extracts. Total carbohydrate content was determined using the phenol-sulfuric acid assay with D-glucose as the standard [26]. Total protein content was determined using the Pierce Bicinchoninic Acid (BCA) Protein Assay Kit (Thermo Scientific, USA) with bovine serum albumin (BSA) as the standard. Total phenolic content was determined by using the Folin-Ciocalteu reagent according to the protocol by Tan and Lim [27], with gallic acid as the standard.

### 2.4. Cell Culture

The hESC line WA01 was purchased from Wicell Research Institute Inc. (Madison, WI, USA). The hESCs were grown on 6-well culture plates coated with Geltrex in StemMACS™ iPS-Brew XF medium supplemented with 1% (v/v) Penicillin-Streptomycin solution. After hESCs reached 80 - 90% confluency, undifferentiated colonies were routinely passaged by enzymatic dissociation into smaller cell clumps using 2 mg/ml collagenase. The cells were grown in a humidified incubator at 37°C with a 5% CO_2_ atmosphere.

#### 2.4.1. Induction of hESC into NSC

Confluent hESC cultures were passaged as small clumps on 6-well culture plates coated with Geltrex. The seeding density in each well was around 15 - 25% confluency. Induction into NSCs was started approximately 24 h after seeding of hESC by changing to Neural Induction Medium (Neurobasal medium supplemented with 2% (v/v) Neural Induction Supplement) with 1% (v/v) Penicillin-Streptomycin solution. The cells were cultured in a humidified incubator at 37°C with 5% CO_2_ for 7 days until confluent. The NSCs were passaged on 6-well culture plates coated with Geltrex by enzymatic dissociation using StemPro Accutase Cell Dissociation Reagent. The cells were resuspended in Neural Expansion Medium (Neurobasal medium and Advanced DMEM/F-12 supplemented with 2% (v/v) Neural Induction Supplement) with 1% (v/v) Penicillin-Streptomycin solution and plated at a density of 3 x 10^5^ - 4 x 10^5^ cells per well. On Day 1 of passaging, ROCK inhibitor Y27632 was added to the medium at a final concentration of 10 *μ*M to prevent cell death. Once NSCs reached 90 – 100% confluency, cells were then routinely passaged. At the 5^th^ passage, NSCs were used in the experiments and differentiated into mature neurons (MN). For the experiments, NSCs were plated on 24-well culture plates coated with Geltrex at a density of 1 x 10^5^ cells per well in Neural Expansion Medium. NSCs were subjected to various treatments after growing for 48 h.

#### 2.4.2. Differentiation of NSC into MN

The NSCs were seeded on 24-well culture plates coated with poly-L-ornithine (20 *μ*g/ml) and laminin (10 *μ*g/ml) at a density of 3 x 10^4^ cells per well in StemPro NSC SFM medium. StemPro NSC SFM medium consisted of KnockOut DMEM/F-12 supplemented with 2 mM GlutaMAX-I Supplement, 20 ng/ml bFGF, 20 ng/ml EGF, and 2% (v/v) StemPro Neural Supplement with 1% (v/v) Penicillin-Streptomycin solution. After 48 h, the medium was replaced with Neural Differentiation Medium (Neurobasal medium supplemented with 2% (v/v) B-27 Serum-Free Supplement and 2 mM GlutaMAX-I Supplement) with 1% (v/v) Penicillin-Streptomycin solution. After 7 days of differentiation, 0.5 mM dibutyryl cAMP was added to the differentiation medium for 3 days to accelerate differentiation into MNs. After 10 days of differentiation, MNs were utilized for experiments.

#### 2.4.3. SH-SY5Y Cell Culture and Differentiation

Human neuroblastoma SH-SY5Y (ATCC CRL-2266) cells were grown in T-75 cell culture flasks with DMEM supplemented with 10% (v/v) FBS and 1% (v/v) Penicillin-Streptomycin solution in a humidified incubator at 37°C with 5% CO_2_. Upon reaching 80 - 90% confluency, the cells were passaged with enzymatic dissociation in 0.25% (w/v) Trypsin-EDTA solution. For differentiation, SH-SY5Y cells were plated on 24-well culture plates at a density of 5 x 10^4^ cells per well in DMEM supplemented with 10% (v/v) FBS and 1% (v/v) Penicillin-Streptomycin solution. After 48 h, the culture medium was changed to Neural Differentiation Medium supplemented with 10 *μ*M RA to induce differentiation and promote the neuronal phenotype. After 5 days of differentiation, SH-SY5Y cells were utilized in the experiments.

### 2.5. Immunostaining for Detection of Neuronal Markers

The NSCs, MNs, and differentiated SH-SY5Y cells were cultured on 4-well chamber slides and fixed with 4% (w/v) paraformaldehyde (PFA) for 15 min at room temperature. Blocking buffer containing 0.1% (w/v) Triton X-100 and 1% (w/v) BSA in PBS was added and incubated for 60 min at room temperature. NSCs were incubated overnight at 4°C with mouse anti-Nestin primary antibody (sc-23927, Santa Cruz Biotechnology Inc., Santa Cruz, CA, USA) diluted 1:100 in blocking buffer. MNs and differentiated SH-SY5Y cells were incubated overnight at 4°C with mouse anti-DCX primary antibody (sc-271390, Santa Cruz Biotechnology Inc.) and mouse anti-TUJ-1 primary antibody (sc-58888, Santa Cruz Biotechnology Inc.) diluted 1:50 in blocking buffer. After overnight incubation, the cells were incubated with goat anti-mouse IgG-fluorescein isothiocyanate (FITC) secondary antibody (sc-2010, Santa Cruz Biotechnology Inc.) and rabbit anti-mouse IgG-phycoerythrin (PE) secondary antibody (sc-358926, Santa Cruz Biotechnology Inc.) diluted 1:100 in blocking buffer for 2 h at room temperature in the dark. The cell nuclei were stained with 4'-6-Diamidino-2-phenylindole (DAPI) (Sigma). Finally, the slide was mounted using Fluoromount Aqueous Mounting Medium (Sigma) and observed under a Nikon Eclipse Ti-S inverted fluorescence microscope. Images were captured using Nikon NIS-Elements microscope imaging software and brightness and contrast were adjusted using ImageJ software (National Institutes of Health, Bethesda, Maryland, USA, https://imagej.nih.gov/ij/).

### 2.6. Cell Viability Assay

For screening of* L. rhinocerus *sclerotial extracts, NSCs, MNs, and differentiated SH-SY5Y cells were plated on 24-well plates, according to the cell densities mentioned previously. The cells were treated with 1 – 1000 *μ*g/ml of* L. rhinocerus *sclerotial HA, CA, RT, or ME extracts for 24 h at 37°C in 5% CO_2_. To determine the effect of heating, each extract (1000 *μ*g/ml) was boiled for 20 min and incubated with the cells for 24 h. The NSCs, MNs, and differentiated SH-SY5Y cells were also exposed to DEX (1 – 1000 *μ*M) for 48 h at 37°C in 5% CO_2_. To investigate the neuroprotective activity of* L. rhinocerus *sclerotial extracts against DEX-induced toxicity, NSCs were coincubated with DEX (1, 100, and 500 *μ*M) and* L. rhinocerus* HA (10 and 100 *μ*g/ml) or ME (5 and 50 *μ*g/ml) extracts for 48 h at 37°C in 5% CO_2_. Cells cultured in medium without* L. rhinocerus *sclerotial extract and DEX served as the negative control.

Cell viability after treatment was measured using the CellTiter-Blue Cell Viability Assay (Promega Inc., Madison, WI, USA) following the manufacturer's protocol. Briefly, the CellTiter-Blue Reagent was added to the culture medium in each well at a ratio of 1:5 (v/v). Cells were then incubated for 1.5 h in the incubator at 37°C in 5% CO_2_. Fluorescence of treated and untreated (negative control) cells and blank wells containing culture medium without cells were measured at a wavelength of 560/590 nm (excitation/emission) using a Tecan infinite M200 Pro microplate reader (Tecan Inc., Maennedorf, Switzerland). Cell viability was calculated as the ratio of average fluorescence readings of the treated cells to that of the negative control cells, after subtracting the average readings from blank wells. The cell viability data was presented as a percentage relative to the negative control.

### 2.7. Neurite Outgrowth Assay and Quantification

The NSCs were plated on 12-well plates coated with poly-L-ornithine and laminin at a density of 3 x 10^4^ cells per well in StemPro NSC SFM medium and incubated for 48 h at 37°C in 5% CO_2_. The NSCs were treated with varying concentrations of* L. rhinocerus* HA or ME extracts (1 - 100 *μ*g/ml) in Neurobasal medium supplemented with 2 mM GlutaMAX-I supplement and 1% (v/v) Penicillin-Streptomycin solution to determine the optimal concentration that induced maximal neurite outgrowth. In addition, NSCs were treated with DEX (1 - 100 *μ*M) to study the GC-induced effect on neurite outgrowth. Cells cultured in medium without* L. rhinocerus *sclerotial extract or DEX served as the negative control, and cells grown in medium with 2% (v/v) B-27 supplement were the positive control. Cells were incubated for 72 h at 37°C in 5% CO_2_ and assessed for neurite outgrowth.

For the quantification of neurite outgrowth, five random fields from each well were photographed and examined for cells with neurite outgrowth, i.e., cells with axon-like extensions that were at least twice the length of the cell body diameter by blind analysis [14]. The total number of cells with neurites and total number of viable cells per well were counted in the ImageJ software. The percentage of neurite-bearing cells was obtained by calculating the ratio of neurite-bearing cells to the total viable cell number in each well.

To further evaluate the neurite outgrowth and extension of NSCs, the cells were plated in 8-well chamber slides and treated with the optimal concentration of* L. rhinocerus* HA and ME extracts as well as DEX (1 – 100 *μ*M) for 72 h. The NSCs were then stained with mouse anti-TUJ-1 primary antibody and goat anti-mouse IgG-FITC secondary antibody following the aforementioned protocol for immunostaining. The slide was mounted and observed under a Nikon Eclipse Ti-S inverted fluorescence microscope and images were captured using the Nikon NIS-Elements microscope imaging software.

### 2.8. Cell Proliferation Assay

The NSCs were plated in 8-well chamber slides and exposed to DEX (1 – 1000 *μ*M) for 48 h at 37°C in 5% CO_2_. Cells cultured in medium without DEX served as the negative control. Next, 10 *μ*M of BrdU was added to the cell culture and incubated overnight at 37°C in 5% CO_2_. After incubation with BrdU, NSCs were fixed with 4% (w/v) PFA for 15 min at room temperature and then treated with 2 M hydrochloric acid for 30 min at room temperature. Next, blocking buffer containing 0.1% (w/v) Triton X-100 and 1% (w/v) BSA in PBS was added to the cell culture and incubated for 60 min at room temperature. Cells were then incubated overnight at 4°C with mouse anti-BrdU primary antibody (sc-32323, Santa Cruz Biotechnology Inc.) diluted 1:50 in blocking buffer. After overnight incubation, cells were incubated with goat anti-mouse IgG-PE secondary antibody diluted 1:100 in blocking buffer for 2 h at room temperature in the dark. Finally, cell nuclei were stained with DAPI and the slide was mounted and observed under a Nikon Eclipse Ti-S inverted fluorescence microscope. Images were captured using the Nikon NIS-Elements microscope imaging software.

For quantification of cell proliferation, five random fields from each well were photographed and the total numbers of viable cells (DAPI-stained) and BrdU-positive cells (PE-stained) were determined using blind analysis. The percentage proliferation was calculated as the ratio of BrdU-positive cells to the total number of viable cells in each well and expressed as percentage relative to the negative control.

### 2.9. Annexin V Flow Cytometry Analysis

The NSCs were seeded on 24-well plates coated with Geltrex in Neural Expansion Medium following the cell seeding density mentioned previously. The NSCs were then cotreated with* L. rhinocerus* ME extract (5 or 50 *μ*g/ml) and DEX (1 or 100 *μ*M) for 48 h at 37°C in 5% CO_2_. Cells cultured in medium without* L. rhinocerus *sclerotial extract and DEX served as the negative control. The percentage of apoptotic cells was determined using the ApopNexin™ FITC Apoptosis Detection Kit (Chemicon, Merck, Germany) following the manufacturer's protocol. Briefly, NSCs were harvested using StemPro Accutase Cell Dissociation Reagent and washed once with ice-cold PBS. After removing PBS, cells were resuspended in ice-cold 1X Binding Buffer and stained with Annexin V conjugated to FITC (Annexin V/FITC) and propidium iodide (PI) for 15 min at room temperature in the dark. Percentages of live cells (Annexin V/FITC- and PI-negative), apoptotic cells (Annexin V/FITC-positive), and necrotic cells (PI-positive) were determined by flow cytometry (BD FACSCalibur, BD Biosciences, USA) and data was analyzed using BD CellQuest Pro Software. The apoptosis level of NSCs was presented as a percentage relative to the negative control.

### 2.10. Hoechst Staining

The NSCs were seeded in 8-well chamber slides and coincubated with* L. rhinocerus* ME extract (5 or 50 *μ*g/ml) and 100 *μ*M DEX or 10 *μ*M etoposide (Santa Cruz Biotechnology Inc.) for 48 h at 37°C in 5% CO_2_. Cells cultured in medium without* L. rhinocerus *sclerotial extract, DEX and etoposide served as the negative control. Etoposide was added as a positive control to induce apoptosis in NSCs. After treatment, NSCs were fixed with 4% (w/v) PFA for 15 min at room temperature and then incubated with PBS containing 0.1% (w/v) Triton X-100 for 15 min at room temperature. Next, 5 *μ*g/ml Hoechst 33342 (Thermo Scientific Inc.) was added to the cells and incubated for 10 min at room temperature in the dark. The slide was then mounted and observed under a Nikon Eclipse Ti-S inverted fluorescence microscope. Images were captured using the Nikon NIS-Elements microscope imaging software.

Cells showing condensation and fragmentation of nuclei were considered apoptotic. For quantification of apoptotic nuclei, five random fields from each well were photographed and the number of total cells and cells with condensed or fragmented nuclei were determined by blind analysis. The percentage of apoptotic nuclei was calculated as the ratio of cells with condensed or fragmented nuclei to the total number of cells in each well.

### 2.11. Western Blot Analysis

The NSCs were plated on 24-well plates coated with Geltrex in Neural Expansion Medium following the cell seeding density mentioned previously. Cells were cotreated with* L. rhinocerus* ME extract (5 or 50 *μ*g/ml) and DEX (1 or 100 *μ*M) for 48 h at 37°C in 5% CO_2_. Cells cultured in medium without* L. rhinocerus *sclerotial extract and DEX served as the negative control. After treatment, cells were lysed in ice-cold RIPA lysis buffer supplemented with protease inhibitor (Thermo Scientific) for 30 min on ice. After centrifugation, cell lysates were transferred to new 1.5 ml tubes and kept at -80°C. Protein concentration was determined using the Pierce BCA Protein Assay Kit. Protein samples were mixed with loading buffer and denatured at 95°C for 10 min. For sodium dodecyl sulfate polyacrylamide gels (SDS-PAGE), equal amounts of each protein sample (20 *μ*g) were loaded along with a prestained protein ladder (CSL-BBL, Cleaver Scientific, UK). Proteins were separated by 10% (w/v) SDS-PAGE and then transferred to nitrocellulose membranes. Ponceau S staining (Santa Cruz Biotechnology Inc.) was performed to assess quality of transfer and to ensure equal loading of total protein. The blot was blocked by using 5% (w/v) nonfat skimmed milk in TBST (Tris-Buffered Saline with 0.05% (v/v) Tween 20) for 1 h at room temperature. The blot was probed with anti-phospho-Akt (pAkt) antibody (Ser473) (1:1000, 9271, Cell Signaling Technology, USA) and anti-*β*-Actin antibody (C4) (1:200, sc-47778, Santa Cruz Biotechnology Inc.) diluted in 5% (w/v) BSA in TBST at 4°C overnight. The blot was then washed three times with TBST and incubated for 1 h at room temperature with secondary antibodies conjugated with horseradish peroxidase (HRP): goat anti-rabbit IgG-HRP (1:5000, sc-2030, Santa Cruz Biotechnology Inc.) and goat anti-mouse IgG (H+L) HRP (1:5000, 62-6520, Invitrogen, USA) diluted in 2.5% (w/v) nonfat skimmed milk in TBST. The blot was then washed five times with TBST, incubated with enhanced chemiluminescence (ECL) substrate (Thermo Scientific) for 5 min at room temperature, and visualized using the ImageQuant LAS 500 imager (GE Healthcare, USA).

Optical density of each band and total protein (Ponceau S staining) was quantified by using the Image Studio Lite software (LI-COR Biosciences) following automated background subtraction. The optical density of the *β*-actin band (loading control) was first standardized to that of total protein, and the optical density of the pAkt band was normalized to optical density of the corresponding *β*-actin band. Results were expressed as the relative changes of pAkt band intensity in the treated cells with respect to the negative control.

### 2.12. Statistical Analysis

All experiments were conducted at least three times and the data was expressed as mean ± standard error mean (SEM). Statistical analysis was performed using GraphPad Prism 5 software. One-way or two-way analysis of variance (ANOVA) followed by Bonferroni's multiple comparison post hoc test was carried out.* P*< 0.05 was considered statistically significant.

## 3. Results

### 3.1. Validation of the* In Vitro* Models

The neural phenotypes of all the* in vitro* models used in this study, namely the human embryonic stem cell (hESC)-derived NSCs and MNs as well as differentiated SH-SY5Y cells, were characterized and validated by immunostaining and fluorescence microscopy for the expression of the relevant neuronal markers (Figure S1). The NSCs derived from hESCs were shown to express Nestin (Figure S1A), an intermediate filament protein which is mainly present in the neural progenitor cells [28]. The MNs differentiated from NSCs (Figure S1B) and the SH-SY5Y cells differentiated with RA (Figure S1C) expressed neuronal markers *β*3-tubulin (TUJ-1) and doublecortin (DCX). TUJ-1 is a class of tubulin family that is specifically found in neurons while DCX is a microtubule-associated protein highly expressed in differentiating neurons [28, 29]. Our previous study has also reported the expression of neural markers in the* in vitro* models: NSCs expressed Nestin and early neural differentiation markers such as Pax6 and Musashi1, while MNs and differentiated SH-SY5Y cells expressed DCX and mature neural differentiation markers such as MAP2 (microtubule-associated protein 2), NCAM (neural cell adhesion molecule), and NFM (neural filament protein - medium) [30]. Taken together, the immunostaining and quantitative real time-polymerase chain reaction (qRT-PCR) analyses of the neural marker expression were able to validate the neural phenotypes of all the* in vitro* models used in this study [30].

### 3.2. Yields and Chemical Compositions of* L. rhinocerus *Sclerotial Extracts

The yields (based on dry weight) and chemical compositions (Table 1) were compared among the different extracts of* L. rhinocerus* sclerotium: hot aqueous extract, cold aqueous extract, room temperature aqueous extract, and methanol extract. Among the extracts,* L. rhinocerus *HA extract had the highest yield (51.5%), both* L. rhinocerus* CA and RT extracts had similar yields (21.6% and 24.3% respectively) and* L. rhinocerus* ME extract had the lowest yield (7.3%). One-way ANOVA showed that the carbohydrate, protein, and phenolic levels among the different* L. rhinocerus *sclerotial extracts differed significantly [carbohydrate: F (3, 8) = 50.57,* P* < 0.0001; protein: F (3, 8) = 30.88,* P* < 0.0001; phenolic: F (3, 8) = 44.20,* P* < 0.0001]. Post hoc Bonferroni test revealed that the* L. rhinocerus* HA extract contained significantly higher carbohydrate content (*P* < 0.001) and significantly lower protein level (*P* < 0.01) than the CA, RT, and ME extracts. There were no significant differences between carbohydrate, protein, and phenolic contents of* L. rhinocerus* CA extract versus RT extract. On the other hand, the* L. rhinocerus* ME extract contained significantly higher phenolic content than all the aqueous extracts (*P* < 0.01).

### 3.3. Effect of* L. rhinocerus *Sclerotial Extracts on Viability of* In Vitro* Models

**Figure 1:**
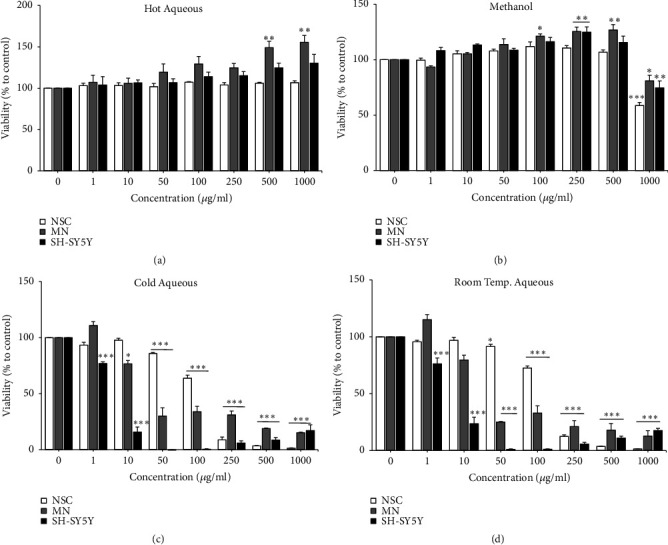
Effects of* L. rhinocerus *(a) HA, (b) ME, (c) CA, and (d) RT extracts (1 - 1000 *μ*g/ml) on the viability of NSCs, MNs, and differentiated SH-SY5Y cells after 24 h of incubation. Results are expressed as mean ± SEM (n = 3). Asterisks denote significant differences compared to 0 *μ*g/ml (negative control); *∗ P *< 0.05, *∗∗ P *< 0.01, *∗∗∗ P *< 0.001.

The sclerotial extracts of* L. rhinocerus* (1 - 1000 *μ*g/ml) were screened for their effect on the viability of the* in vitro* models (Figure 1). Two-way ANOVA indicated that the different concentrations of* L. rhinocerus* aqueous and methanol sclerotial extracts had significant effects on the viability of all* in vitro* models [*L. rhinocerus* HA: F (14, 48) = 2.462,* P* < 0.05;* L. rhinocerus* ME: F (14, 48) = 3.251,* P* < 0.05;* L. rhinocerus* CA: F (14, 48) = 62.51,* P* < 0.0001;* L. rhinocerus* RT: F (14, 48) = 51.62,* P* < 0.0001]. Both* L. rhinocerus* HA extract at all concentrations tested (Figure 1(a)) and ME extract at concentrations ranging from 1 - 500 *μ*g/ml (Figure 1(b)) did not decrease the viability of NSCs, MNs, and differentiated SH-SY5Y cells. However, both* L. rhinocerus* CA (Figure 1(c)) and RT (Figure 1(d)) extracts significantly reduced the viability of all* in vitro* models in a dose-dependent manner (*P* < 0.05,* P* < 0.001). These results indicated that* L. rhinocerus* HA and ME extracts (at concentrations up to 500 *μ*g/ml) are noncytotoxic to NSCs, MNs, and differentiated SH-SY5Y cells, whereas* L. rhinocerus* CA and RT extracts are cytotoxic.

Further boiling of the* L. rhinocerus *sclerotial extracts (1000 *μ*g/ml) was conducted to compare the effects of heat-treated extracts on the viability of the* in vitro* models (Figure S2). Two-way ANOVA demonstrated that boiled* L. rhinocerus* HA extract had no significant effect [F (2, 12) = 0.3872,* P* > 0.05] on the viability of the* in vitro* models (Figure S2A), whereas boiled* L. rhinocerus* ME, CA, and RT extracts displayed significant effects on the viability of all* in vitro* models [boiled ME: F (2, 12) = 8.564,* P* < 0.01; boiled CA: F (2, 12) = 15.46,* P* < 0.001; boiled RT: F (2, 12) = 24.35,* P* < 0.0001]. Both boiled* L. rhinocerus* CA (Figure S2C) and RT (Figure S2D) extracts significantly increased the viability (*P* < 0.001) of all* in vitro* models, whereas boiled* L. rhinocerus* ME extract significantly increased the viability of differentiated SH-SY5Y cells only (*P* < 0.001) (Figure S2B). These results indicated that boiling of* L. rhinocerus* CA, RT, and ME extracts (1000 *μ*g/ml) could reverse their cytotoxic effects on the* in vitro* models.

### 3.4. Effects of* L. rhinocerus* HA and ME Extracts on Neurite Outgrowth of NSCs

**Figure 2:**
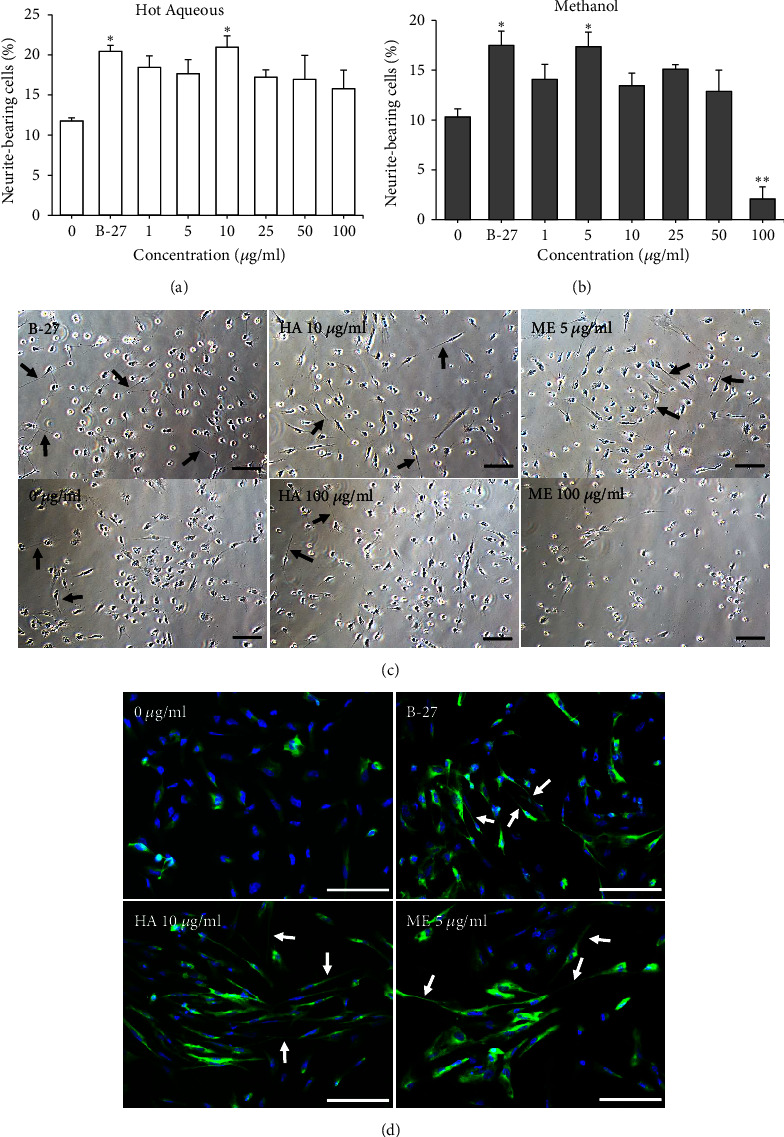
Effects of* L. rhinocerus *(a) HA and (b) ME extracts (1 - 100 *μ*g/ml) on neurite outgrowth of NSCs after 72 h of incubation. Results are expressed as mean ± SEM (n = 4). Asterisks denote significant differences compared to 0 *μ*g/ml (negative control); *∗ P *< 0.05, *∗∗ P *< 0.01. (c) Images of NSCs treated with 0 *μ*g/ml, B-27 supplement,* L. rhinocerus *HA extract (10 and 100 *μ*g/ml) and ME extract (5 and 100 *μ*g/ml). (d) Immunostaining and fluorescence microscopy of TUJ-1 expression in NSCs treated with 0 *μ*g/ml, B-27 supplement,* L. rhinocerus *HA extract (10 *μ*g/ml) and ME extract (5 *μ*g/ml). Nuclei were stained with DAPI (blue) and neurites were stained with TUJ-1 (green). Arrows indicate extension of neurites that was at least twice the length of the cell body diameter. Scale bar: 100 *μ*m.

Both noncytotoxic* L. rhinocerus* HA and ME extracts (at a low concentration range, 1 - 100 *μ*g/ml) were further tested for neurite outgrowth assay using hESC-derived NSCs (Figure 2). As the positive control, B-27 supplement significantly increased the percentage of neurite-bearing cells (*P* < 0.05) compared to the negative control. One-way ANOVA revealed that* L. rhinocerus* HA and ME extracts had significant effects on the neurite outgrowth of NSCs [*L. rhinocerus* HA: F (7, 24) = 2.895,* P* < 0.05;* L. rhinocerus* ME: F (7, 24) = 13.02,* P* < 0.0001]. The* L. rhinocerus* HA extract at 10 *μ*g/ml (Figure 2(a)) and ME extract at 5 *μ*g/ml (Figure 2(b)) induced maximal neurite outgrowth in NSCs, as shown by the significantly increased percentage of neurite-bearing cells compared to the negative control (*P* < 0.05). The percentage of neurite-bearing cells in NSCs treated with 10 *μ*g/ml* L. rhinocerus* HA extract or 5 *μ*g/ml* L. rhinocerus* ME extract was also comparable to that of B-27 treatment (positive control).

Immunostaining and fluorescence microscopy were performed to examine TUJ-1 expression in NSCs treated with 10 *μ*g/ml* L. rhinocerus* HA extract or 5 *μ*g/ml* L. rhinocerus* ME extract (i.e., at concentrations that promoted maximal neurite outgrowth). Neurite extension of twice the length of the cell body diameter was observed along with the expression of TUJ-1 in NSCs treated with* L. rhinocerus* HA and ME extracts (Figure 2(d)). Taken together, these results showed that* L. rhinocerus* HA (10 *μ*g/ml) and ME (5 *μ*g/ml) extracts stimulated maximal neurite outgrowth in NSCs and suggested that both extracts potentially contain neuroactive compounds that promote neuritogenesis. Therefore,* L. rhinocerus* HA and ME extracts were further tested in neuroprotective assays against DEX-induced effects on hESC-derived neural lineages.

### 3.5. Effects of DEX on the Viability of* In Vitro* Models

**Figure 3:**
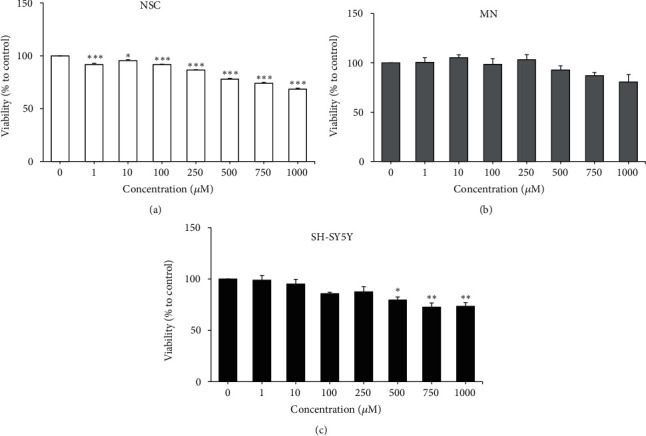
Effects of DEX (1 - 1000 *μ*M) on cell viability of (a) NSCs, (b) MNs, and (c) differentiated SH-SY5Y cells after 48 h of incubation. Results are expressed as mean ± SEM (n = 3 - 4). Asterisks denote significant differences compared to 0 *μ*M (negative control); *∗ P *< 0.05, *∗∗ P *< 0.01, *∗∗∗ P *< 0.001.

Screening of DEX (1 - 1000 *μ*M) for its effects on the viability of the* in vitro* models was conducted (Figure 3). One-way ANOVA revealed that DEX had significant effects on the viability of all* in vitro* models [NSCs: F (7, 16) = 202.3,* P* < 0.0001; MNs: F (7, 24) = 3.320,* P* < 0.05; differentiated SH-SY5Y cells: F (7, 16) = 8.918,* P* < 0.001]. Post hoc Bonferroni test showed that DEX significantly reduced viability of NSCs (*P* < 0.05,* P* < 0.001) at all concentrations tested in a dose-dependent manner (Figure 3(a)). On the other hand, DEX significantly reduced the viability of differentiated SH-SY5Y cells (*P* < 0.05,* P* < 0.01) at high concentrations (500, 750, and 1000 *μ*M) (Figure 3(c)). No significant differences were observed on the viability of MNs at all concentrations of DEX tested (Figure 3(b)). These results indicated that NSCs were more susceptible to DEX compared to both MNs and SH-SY5Y cells, which were differentiated and expressed more mature neuronal phenotypes. Therefore, hESC-derived NSCs were selected as the* in vitro* model for studying the DEX-induced effects and neuroprotective assays to test the* L. rhinocerus *sclerotial extracts.

### 3.6. Effects of DEX on the Proliferation and Neurite Outgrowth of NSCs

**Figure 4:**
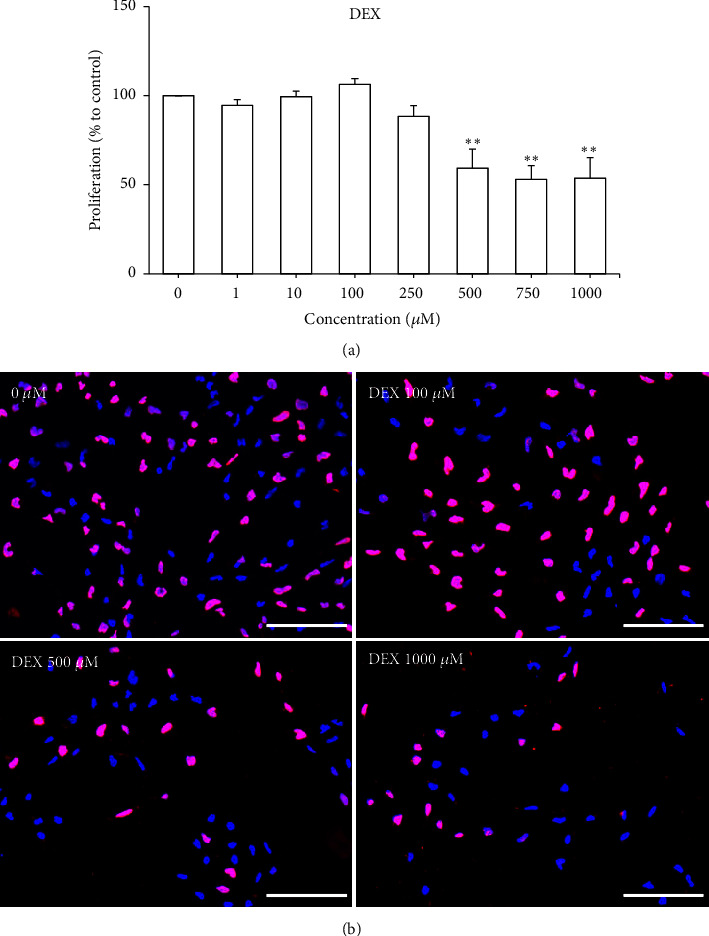
Effects of DEX (1 - 1000 *μ*M) on (a) proliferation of NSCs after 48 h of treatment. Results are expressed as mean ± SEM (n = 4). Asterisks denote significant differences compared to 0 *μ*M (negative control); *∗∗ P *< 0.01. (b) Immunostaining and fluorescence microscopy of NSCs with incorporation of BrdU in the nuclei. Nuclei were stained with DAPI (blue) while BrdU-positive nuclei were stained pink. Scale bar: 100 *μ*m.

Screening of DEX (1 - 1000 *μ*M) for its effects on proliferation of NSCs was carried out to examine whether the DEX-induced reduction in cell viability was related to decreased proliferation. One-way ANOVA demonstrated that DEX had a significant effect on the proliferation of NSCs [F (7, 24) = 11.15,* P* < 0.0001]. High concentrations of DEX (500, 750, and 1000 *μ*M) caused significant decreases in the proliferation of NSCs (*P* < 0.01) (Figure 4(a)). Post hoc Bonferroni tests showed that DEX at a concentration range of 1 - 250 *μ*M had no significant effect on the proliferation of NSCs compared to the negative control. Therefore, the observed decrease in viability of NSCs treated with DEX at concentrations lower than 500 *μ*M may not be caused by reduced proliferation, but could be potentially attributed to cell death induced by DEX.

**Figure 5:**
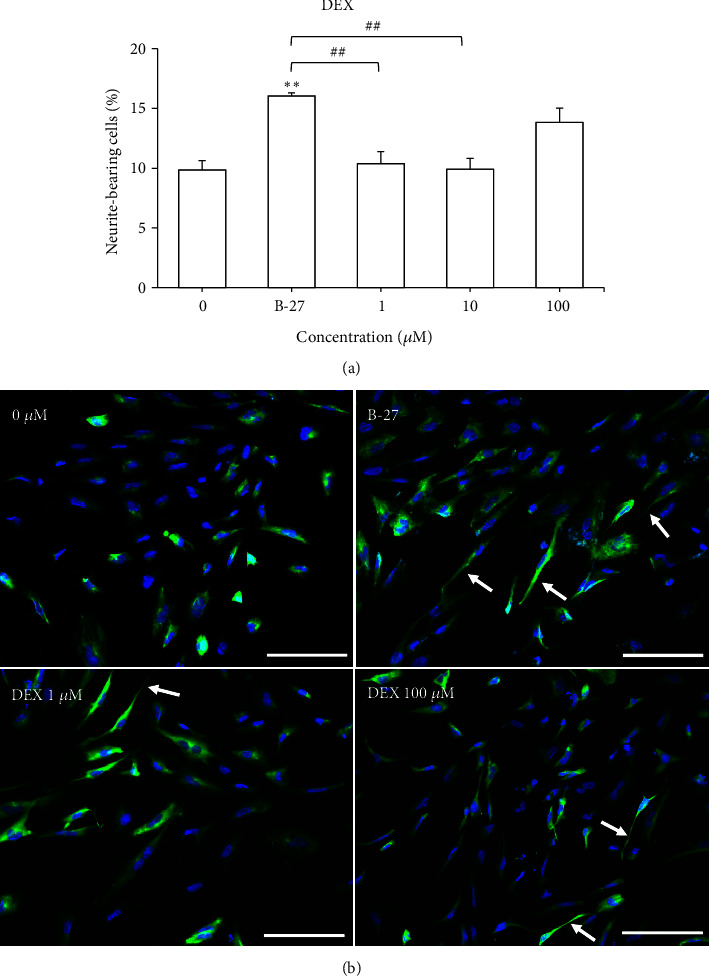
Effects of DEX (1 - 100 *μ*M) on (a) neurite outgrowth of NSCs after 72 h of treatment. Results are expressed as mean ± SEM (n = 4). Asterisks denote significant differences compared to 0 *μ*M (negative control); *∗∗ P *< 0.01. Hashtags denote significant differences compared to B-27 treatment (positive control); ^##^* P *< 0.01. (b) Immunostaining and fluorescence microscopy of TUJ-1 expression in NSCs treated with 0 *μ*M, B-27 supplement and DEX (1 and 100 *μ*M). Nuclei were stained with DAPI (blue) and neurites were stained with TUJ-1 (green). Arrows indicate neurite outgrowth that was at least twice the length of the cell body diameter. Scale bar: 100 *μ*m.

The effects of DEX (1 - 100 *μ*M) on the neurite outgrowth of NSCs were investigated (Figure 5). As a positive control, B-27 supplement significantly increased the percentage of neurite-bearing cells (*P* < 0.01) compared to the negative control. One-way ANOVA showed that there was a significant effect of treatment on the neurite outgrowth of NSCs [F (4, 15) = 10.05,* P* < 0.001]. Treatment with DEX (1 and 10 *μ*M) resulted in significantly lower percentage of neurite-bearing cells in NSCs (*P* < 0.01) compared to the positive control (Figure 5(a)). Post hoc Bonferroni test indicated that DEX (1 - 100 *μ*M) had no significant effect on the percentage of neurite-bearing cells compared to the negative control. In addition, immunostaining and fluorescence microscopy of TUJ-1 expression in NSCs treated with DEX showed less neurite extension compared to NSCs treated with B-27 supplement (Figure 5(b)).

### 3.7. Neuroprotective Effects of* L. rhinocerus* HA and ME Extracts on the Viability of DEX-Treated NSCs

**Figure 6:**
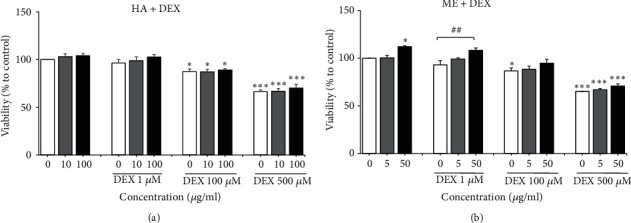
Neuroprotective effects of* L. rhinocerus *(a) HA extract (10 and 100 *μ*g/ml) and (b) ME extract (5 and 50 *μ*g/ml) on the viability of NSCs treated with DEX (1, 100, and 500 *μ*M). Results are expressed as mean ± SEM (n = 4 - 5). Asterisks denote significant differences compared to 0 *μ*g/ml (negative control); *∗ P *< 0.05, *∗∗∗ P *< 0.001. Hashtags denote significant differences compared to cells treated with DEX only; ^##^* P *< 0.01.

As* L. rhinocerus* HA and ME extracts were noncytotoxic and exhibited neuritogenic property, their neuroprotective effects on DEX-induced reduction of NSC viability were investigated. NSCs exposed to DEX (1, 100, and 500 *μ*M) were cotreated with* L. rhinocerus* HA (10 and 100 *μ*g/ml) or ME (5 and 50 *μ*g/ml) extracts (Figure 6). Two-way ANOVA revealed that treatment of* L. rhinocerus* ME extract or DEX alone had significant effects on the survival of NSCs [*L. rhinocerus* ME: F (2, 42) = 14.30,* P* < 0.0001; DEX: F (3, 42) = 90.41,* P* < 0.0001], whereas* L. rhinocerus* HA extract had no significant effect on the viability of NSCs [F (2, 34) = 1.986,* P* > 0.05]. Two-way ANOVA also demonstrated no significant interaction effects between* L. rhinocerus* HA or ME extracts and DEX. DEX significantly decreased viability of NSCs in a dose-dependent manner (*P* < 0.05,* P* < 0.001), whereas treatment with 50 *μ*g/ml* L. rhinocerus* ME extract significantly increased viability of NSCs compared to the negative control (*P* < 0.05). Coincubation of* L. rhinocerus* HA (10 and 100 *μ*g/ml) extract with DEX (1, 100, and 500 *μ*M) did not significantly increase viability of NSCs (Figure 6(a)). However, coincubation of 50 *μ*g/ml* L. rhinocerus* ME extract with 1 *μ*M DEX significantly increased viability of NSCs (*P* < 0.01) (Figure 6(b)). These results suggested that* L. rhinocerus* ME extract might exert neuroprotective effects on NSCs against DEX. The neuroprotective effects of* L. rhinocerus* ME extract against DEX-induced cytotoxic effects in NSCs were further investigated by apoptosis assays and Western blot analysis.

### 3.8. Neuroprotective Effect of* L. rhinocerus* ME Extract against DEX-Induced Apoptosis in NSCs

**Figure 7:**
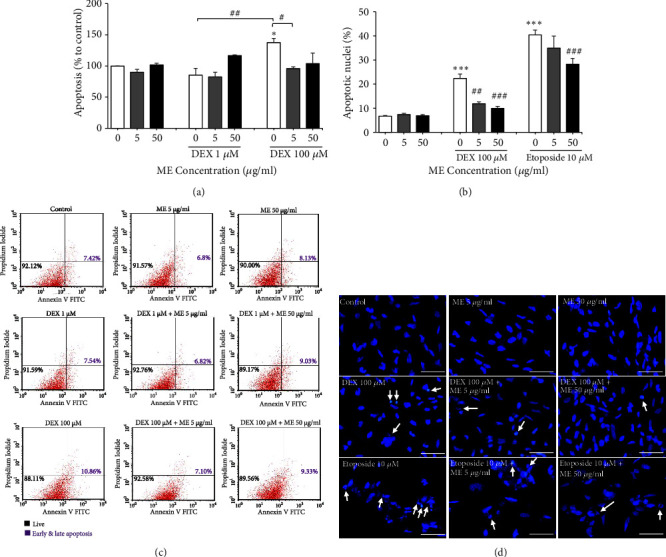
Neuroprotective effects of* L. rhinocerus *ME extract (5 and 50 *μ*g/ml) on (a) apoptosis of NSCs treated with DEX (1 and 100 *μ*M) and (b) percentage of apoptotic nuclei in NSCs treated with 100 *μ*M DEX and 10 *μ*M etoposide. Results are expressed as mean ± SEM (n = 3 - 4). Asterisks denote significant differences compared to 0 *μ*g/ml (negative control); *∗ P *< 0.05, *∗∗∗ P *< 0.001. Hashtags denote significant differences compared to cells treated with DEX or etoposide only; ^#^* P *< 0.05, ^##^* P *< 0.01, ^###^* P *< 0.001. (c) Representative quadrants from flow cytometry analysis. The lower left quadrant represents live cells while the lower right and upper right quadrants represent early and late apoptotic cells, respectively. (d) Immunostaining and fluorescence microscopy of NSCs nuclei stained with Hoechst 33342 (blue). Arrows indicate apoptotic cells with condensed or fragmented nuclei. Scale bar: 50 *μ*m.

The neuroprotective effect of* L. rhinocerus* ME extract against DEX-induced apoptosis in NSCs was investigated using Annexin V flow cytometry analysis (Figure 7(a)). Two-way ANOVA demonstrated that cotreatment with* L. rhinocerus* ME extract and DEX had significant effects on the apoptosis of NSCs [F (4, 18) = 4.749,* P* < 0.01]. Post hoc Bonferroni test showed that 100 *μ*M DEX significantly increased apoptosis of NSCs compared to that of the negative control (*P* < 0.05), whereas cotreatment with 5 *μ*g/ml* L. rhinocerus* ME extract and 100 *μ*M DEX significantly decreased the apoptosis of NSCs (*P* < 0.05). Therefore, these results suggested that cotreatment with* L. rhinocerus* ME extract can potentially provide neuroprotection by attenuating DEX-induced apoptosis in NSCs.

The neuroprotective effects of* L. rhinocerus* ME extract against the apoptotic effects of 100 *μ*M DEX were further confirmed using Hoechst staining (Figure 7(b)). Hoechst staining of NSCs cotreated with* L. rhinocerus* ME extract and DEX or etoposide (positive control) showed that nuclei of the negative control cells appeared to be round or elongated with overall similar level of blue fluorescence, whereas the nuclei of apoptotic cells were brighter and smaller in size (chromatin condensation) or fragmented (Figure 7(d)). Two-way ANOVA indicated that there was significant interaction effects in the cotreatment with* L. rhinocerus* ME extract and DEX or etoposide on the percentage of apoptotic nuclei in NSCs [F (4, 27) = 3.575,* P* < 0.05]. Post hoc Bonferroni test showed that 10 *μ*M etoposide or 100 *μ*M DEX significantly increased the percentage of apoptotic nuclei in NSCs (*P* < 0.001), whereas cotreatment with* L. rhinocerus* ME extract (5 or 50 *μ*g/ml) and DEX or etoposide significantly decreased the percentage of apoptotic nuclei in NSCs (*P* < 0.01,* P* < 0.001). These results demonstrated that etoposide and DEX treatments induced apoptosis in NSCs while cotreatment with* L. rhinocerus* ME extract attenuated the apoptotic effects of etoposide and DEX on NSCs.

### 3.9. Neuroprotective Effect of* L. rhinocerus* ME Extract on Phospho-Akt Expression in DEX-Treated NSCs

**Figure 8:**
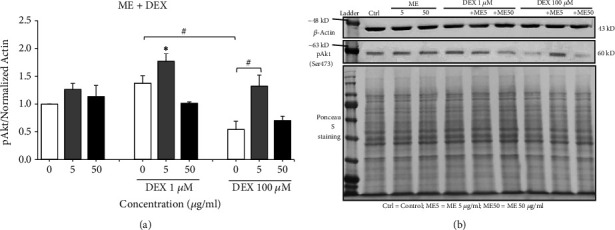
Neuroprotective effects of* L. rhinocerus *ME extract (5 and 50 *μ*g/ml) on (a) pAkt expression levels of NSCs treated with DEX (1 and 100 *μ*M). Results are expressed as mean ± SEM (n = 3 - 4). Asterisks denote significant differences compared to 0 *μ*g/ml (negative control); *∗ P *< 0.05. Hashtags denote significant differences compared to cells treated with DEX only; #* P *< 0.05. (b) Representative Western blot images for *β*-actin, pAkt, and Ponceau S staining. *β*-actin and Ponceau S staining served as the loading controls.

Western blot analysis was conducted to detect changes in the expression level of phospho-Akt (pAkt) in NSCs cotreated with* L. rhinocerus* ME extract and DEX (Figure 8). Two-way ANOVA revealed that the treatment with* L. rhinocerus* ME extract or DEX alone significantly affected the pAkt expression level in NSCs [*L. rhinocerus* ME: F (2, 21) = 13.26,* P* < 0.001; DEX: F (2, 21) = 10.61,* P* < 0.001], but there was no significant interaction effect between the* L. rhinocerus* ME extract and DEX. Treatment of 100 *μ*M DEX decreased the expression level of pAkt compared to the negative control, whereas cotreatment with 5 *μ*g/ml* L. rhinocerus* ME extract and 100 *μ*M DEX significantly increased the pAkt expression level in NSCs (*P* < 0.05) (Figure 8(a)). Taken together, these results showed that 100 *μ*M DEX reduced the pAkt expression level in NSCs, and cotreatment with the* L. rhinocerus* ME extract attenuated the DEX-induced decrease in pAkt expression. This suggests that the Akt signaling pathway may be involved in the neuroprotective effects of* L. rhinocerus* ME extract against DEX-induced apoptosis in NSCs.

## 4. Discussion

This study demonstrated that the aqueous and methanol extracts of* L. rhinocerus* sclerotium have different chemical compositions and bioactivities. Among the extracts,* L. rhinocerus* HA extract had high carbohydrate content, CA and RT extracts had high protein contents, and ME extract had high phenolic content. Both* L. rhinocerus* HA and ME extracts were found to have negligible cytotoxicity and exhibited neuritogenic properties in promoting neurite outgrowth in hESC-derived NSCs. By contrast, both* L. rhinocerus* CA and RT extracts displayed strong cytotoxic effects on all* in vitro* models, and heat treatment of both extracts abolished the cytotoxicity. In addition, DEX at high concentrations was found to decrease NSC proliferation, but had no effect on the neurite outgrowth. Furthermore, our study demonstrated that DEX exerted adverse effects on the viability of NSCs by inducing apoptosis and nuclear morphological changes, such as chromatin condensation and nuclear fragmentation. On the other hand, the* L. rhinocerus* ME extract exhibited potential neuroprotective activities against GC-induced cell death in NSCs. Cotreatment with* L. rhinocerus* ME extract and DEX in NSCs attenuated DEX-induced apoptosis, reduced apoptotic cell nuclei morphology, and reversed DEX-induced decrease in pAkt expression.

### 4.1. Yield and Chemical Compositions of Different* L. rhinocerus *Sclerotial Extracts

The HA extraction of* L. rhinocerus* sclerotium gave the highest yield, followed by CA and RT extractions, whereas ME extraction had the lowest yield. Previous findings reported similar trends in the yields of hot aqueous, cold aqueous, and methanol extracts of* L. rhinocerus* cultivar TM02 sclerotium powder [31, 32]. Aqueous extraction produced higher yields of extracts than methanol extraction, indicating that the sclerotium of* L. rhinocerus* cultivar TM02 contains a higher proportion of water-soluble compounds. Hot aqueous extraction is the traditional and most common method of preparation used by native communities in Malaysia. The sclerotium of* L. rhinocerus* is typically boiled with other herbs to produce a decoction that can boost weak constitution and treat various diseases [7]. This hot aqueous extraction is performed at high temperature (100°C), which explains the high carbohydrate content and low protein level in* L. rhinocerus* HA extract. The application of heat in the hot aqueous extraction method helps to degrade the chitinous cell wall of* L. rhinocerus* and increases the solubility of polysaccharides such as glucans [9]. Additionally, high temperatures also caused the breakdown of thermolabile substances such as proteins or peptides [9]. Similar to previous studies, our* L. rhinocerus* ME extract showed the highest phenolic content and the lowest carbohydrate level among the sclerotial extracts. Mushrooms normally consist of high phenolic content which are secondary metabolites that exert strong antioxidant effects [32]. The methanol extraction method used to extract the* L. rhinocerus* sclerotium utilized aqueous methanol (80% methanol and 20% water), which can broaden the range of extracted compounds, particularly components with high polarity such as phenolic compounds [33]. Carbohydrates such as dietary fibers have been reported to be the main constituent of* L. rhinocerus* sclerotium, but they are insoluble in methanol during methanol extraction. However, simpler components such as amino acids, sugars, and peptides are still soluble in aqueous methanol [33].

### 4.2. Effects of* L. rhinocerus *Sclerotial Extracts on Viability of* In Vitro* Models

The negligible cytotoxicity of* L. rhinocerus* hot aqueous and methanol sclerotial extracts has been demonstrated in both cancer and normal cell lines [9, 32, 33]. On the other hand, the cytotoxic effects of cold aqueous extract of the* L. rhinocerus* sclerotium were reported in several human cancer cell lines including lung, breast, prostate, and colorectal carcinoma cells [9, 32, 34]. However, there have been contradictory findings regarding the cytotoxicity of* L. rhinocerus* cold aqueous extract on normal cell lines [9, 34]. In our study, the* L. rhinocerus* CA and RT extracts exhibited nonselective cytotoxicity against normal and cancer human neuronal cell lines. The cytotoxic agents in both* L. rhinocerus* CA and RT extracts have been postulated to be heat-sensitive compounds, as demonstrated by the loss of cytotoxicity with heat treatment and the negligible cytotoxicity of* L. rhinocerus* HA extract extracted at high temperature. A previous study also demonstrated the loss of cytotoxicity of cold aqueous sclerotial extract of* L. rhinocerus* after being heated to 80 and 100°C [9]. The heat-labile proteins present in the* L. rhinocerus* cold aqueous extract were found to be potentially cytotoxic as proteins denatured at temperatures higher than 60°C [9]. Furthermore, cytotoxic proteins from the medium-molecular-weight fraction of* L. rhinocerus* cold aqueous extract were reported to have selective cytotoxicity towards human breast cancer cells and were subsequently isolated and identified as subtilisin-like serine protease [35]. Taken together, our findings suggest the presence of similar heat-labile proteins in the* L. rhinocerus* CA and RT extracts that exhibited cytotoxic effects on all* in vitro* models used in this study.

### 4.3. *L. rhinocerus* HA and ME Extracts Promoted Neurite Outgrowth of NSCs

Our study found that* L. rhinocerus* HA (10 *μ*g/ml) and ME (5 *μ*g/ml) extracts at low concentrations were able to promote maximal neurite outgrowth in NSCs. Similarly, the hot aqueous extract of* L. rhinocerus* sclerotium has been reported to stimulate neurite outgrowth in the brain, retinal, and spinal cord cells from chick embryos as well as mouse neuroblastoma (N2a) and rat pheochromocytoma (PC-12) cells [14, 15, 36, 37]. The ethanolic extract and crude polysaccharides of* L. rhinocerus *sclerotium have also been shown to induce neurite extension in PC-12 cells [37]. The promotion of neurite outgrowth in N2a cells by* L. rhinocerus *sclerotial extract was possibly attributed to the presence of polysaccharides or triterpenoids [15]. Secondary metabolites isolated from extracts of other mushrooms have been reported to potentiate NGF-induced neurite outgrowth in PC-12 cells, including cyathane diterpenoids isolated from methanolic extract of* Sarcodon scabrosus* and hericenones isolated from ethanolic extract of* Hericium erinaceus *[38, 39]. Sclerotium of* L. rhinocerus* was reported to have high carbohydrate content including polysaccharides such as *β*-glucans, which are bioactive components with anticancer and immunomodulatory activities [40]. Furthermore, chromatographic analysis of* L. rhinocerus *aqueous sclerotial extracts and genome sequencing of* L. rhinocerus* sclerotium (cultivar TM02) demonstrated many bioactive secondary metabolites, such as triterpenes, terpenoids, phenolic, and alkaloids [9, 41]. In our study,* L. rhinocerus* HA and ME extracts exhibited neurite stimulatory activity in hESCs-derived NSCs. Our results suggest that the neuroactive compounds present in the* L. rhinocerus *sclerotial extracts could have potential in promoting neurite outgrowth and differentiation of neural progenitor cells into mature neurons. The neuritogenic property of* L. rhinocerus *sclerotial extracts demonstrated in our study could be linked to the presence of high carbohydrate levels in the HA extract and high phenolic contents in the ME extract. This implies that the neuroactive polysaccharides and/or secondary metabolites in the* L. rhinocerus *sclerotial extracts could be used to stimulate neurite outgrowth in NSCs. However, the potential neuroactive compounds present in the* L. rhinocerus *sclerotial extracts need to be further explored and elucidated.

### 4.4. DEX-Induced Effects on the* In Vitro* Models

In this study, DEX at high concentrations was found to reduce the proliferation of NSCs. In contrast to our findings, previous studies reported that DEX at much lower concentrations had adverse effects on cell proliferation of animal- and human-based models. Both* in vitro* and* in vivo* studies reported that treatment with DEX (1 and 5 *μ*M) lowered proliferation and viability of NSCs in rat embryonic and adult brains, indicating the detrimental effects of GC on brain development [16, 42–44]. It was suggested that DEX might affect cell proliferation by promoting ubiquitination of cyclin D1 which controls the proliferation of embryonic NSCs [43]. Additionally, DEX was found to have a negative impact on the proliferation of human neural progenitor cells (hNPC) and ReNcell CX cells (an immortalized hNPC) [18, 45]. Treatment with 1 *μ*M DEX in hNPCs was shown to downregulate Wnt signaling, which is involved in maintenance and proliferation of various types of stem cells [18]. Possible reasons for the differences in our findings of DEX-induced effects on NSC proliferation and findings in other previous studies might be the variability in species or cell types, as well as divergent protocols used in treatments or culturing of cells. On the other hand, DEX did not affect neurite outgrowth of NSCs in our study, which is in agreement with previous studies which reported that DEX had no effect on differentiation of rat embryonic NSCs and hippocampal progenitor cells [16, 43, 44]. Besides, exposure to 30 *μ*M DEX did not affect the neurite outgrowth or the number of neurons in both hESC-derived neural cells (hN2) and primary rat cortical neural cells [46]. However, contradictory results have been reported on the inhibitory action of DEX on the differentiation of SH-SY5Y cells. Exposure to 10 *μ*M DEX was demonstrated to restrict the number and length of neurites in SH-SY5Y cells [47]. It was also reported that DEX impeded the spontaneous differentiation of hNPC to neurons and induced the generation of glial cells [18]. Taken together, this suggests that DEX may exhibit differential effects on neurite outgrowth depending on the type of* in vitro* models used.

### 4.5. Neuroprotective Effect of* L. rhinocerus* ME Extract against DEX-Induced Apoptosis in NSCs

Our study showed that DEX induced apoptosis as demonstrated by nuclear condensation and fragmentation in NSCs. Apoptosis is programmed cell death that is characterized by morphological modifications such as cell shrinkage and chromatin condensation and biochemical features such as DNA fragmentation and by the expression of cell surface markers such as phosphatidylserine [48]. It was reported that DEX caused apoptosis of hippocampal neural progenitor cells and neurons in rat, and cerebellar neuronal cells in mice and rat [49–53]. It was also shown that DEX induced chromatin condensation and nuclear fragmentation in PC-12 cells and promoted caspase-3 activity and reactive oxygen species (ROS) generation in rat hippocampal NPCs [54, 55]. Although bioactivities of* L. rhinocerus* extracts have been reported including anti-inflammatory, anticancer, neuritogenic, and immunomodulatory activities [13], little is known about the neuroprotective effects of* L. rhinocerus* extracts against GC-induced toxicity in neuronal cells. Interestingly, in our study, the* L. rhinocerus* ME extract was observed to have potential neuroprotective effects in NSCs by attenuating DEX-induced apoptosis and cell nuclei apoptotic morphology. Several studies have described that extracts from other medicinal mushroom (e.g.,* Ganoderma lucidum* and* Hericium erinaceus*) had antiapoptotic effects in neuronal cells [56–59]. The Active Hexose Correlated Compound (AHCC) isolated from extracts of different types of basidiomycetes was demonstrated to suppress DEX-induced apoptosis in rat thymocytes [60]. In addition, some studies found that other mushroom extracts were able to reduce ROS production in human neuroblastoma cells (SK-N-SH) and PC-12 cells [57, 61]. Methanol extracts of* L. rhinocerus* sclerotium have been shown to possess antioxidant activities [32, 62]. Our findings that reported high phenolic content in* L. rhinocerus* ME extract suggest the presence of antioxidant activity. Therefore, the neuroprotective effects of the* L. rhinocerus* ME extract against DEX-induced apoptosis in NSCs could potentially be attributed to antioxidants in the extract. The underlying mechanisms of the antioxidant properties of the* L. rhinocerus* ME extract in relation to its neuroprotective activity on the DEX-induced effects in neuronal cells need to be further explored.

Our study also demonstrated that DEX decreased the expression level of pAkt in NSCs, and coincubation with the* L. rhinocerus* ME extract attenuated this DEX-induced decrease in pAkt expression. The increase in the pAkt expression level in NSCs could be linked to the decrease in percentage of apoptosis observed in our study. This suggests that DEX may promote apoptosis in NSCs by suppressing Akt activation whereas* L. rhinocerus* ME extract can potentially exert neuroprotective effects against DEX-induced apoptosis by stimulating Akt phosphorylation. Akt, also known as protein kinase B, is a serine/threonine protein kinase which serves as the main mediator of survival signaling generated via activation of the PI3K pathway [63]. Akt activity can be fully activated via phosphorylation of specific sites such as Thr308 and Ser473, which then promotes cell survival by providing protection against apoptotic cell death [63, 64]. It was reported that DEX promoted apoptosis in chondrocytes by decreasing Akt phosphorylation and suppressing the PI3K signaling pathway [65]. Moreover, corticosterone, a natural GC, was shown to inhibit Akt activation and reduce survival of rat embryonic NSCs [66]. On the other hand, several studies found that Akt activation can inhibit apoptosis in neuronal cells through modulation of caspase activation and expression of anti- and proapoptotic proteins in the Bcl-2 family [67–70]. Taken together, our findings suggested that the* L. rhinocerus* ME extract could potentially promote Akt activation and enhance neuronal resistance to DEX-induced apoptosis in NSCs. However, further research is required to validate the involvement of Akt signaling in the neuroprotective effects of* L. rhinocerus*.

Induction of apoptosis by DEX in NSCs may have critical clinical significance, as treatment using synthetic GCs can cause loss of neural progenitor cells and reduction of specific neuronal populations in children, leading to adverse effects on certain cognitive or motor skills [55]. It was reported that school-age children who received early postnatal DEX therapy for treatment of severe lung disorder exhibited decreased growth rate, impaired motor function, and poor cognitive skills [19]. Therefore, it is important to reduce or prevent DEX-induced adverse effects on neuronal cells. Our study demonstrated the potential neuroprotective effects of* L. rhinocerus* ME extract using the human-derived NSCs, which have higher resemblance of the human neural physiology compared to the animal-derived neuronal models used in previous studies [14, 15, 37]. This study also suggests that* L. rhinocerus* ME extract contains compounds with potential neuroprotective activity against DEX-induced toxicity in NSCs.

## 5. Conclusion

Our study found that different extracts of* L. rhinocerus* sclerotium had opposing effects on the viability of the* in vitro* models. Hot aqueous and methanol extracts of* L. rhinocerus* sclerotium were found to be noncytotoxic in hESC-derived NSCs, MNs, and differentiated SH-SY5Y cells, whereas cold aqueous and room temperature aqueous extracts had strong cytotoxic effects. In addition, hot aqueous and methanol extracts of* L. rhinocerus *sclerotium exhibited neuritogenic property by stimulating neurite outgrowth in NSCs. Elevated levels of the synthetic glucocorticoid, DEX, decreased viability of NSCs by promoting apoptosis and inducing chromatin condensation and nuclear fragmentation, whereas methanol extract of* L. rhinocerus* sclerotium attenuated this DEX-induced apoptosis in NSCs. The* L. rhinocerus* methanol extract was also shown to attenuate the DEX-induced reduction in phospho-Akt level in NSCs, suggesting the involvement of PI3K/Akt signaling pathway in the neuroprotective activity of* L. rhinocerus*. Our study demonstrated the potential of using hESC-derived neuronal cells as* in vitro* models for the screening of neuroprotective activities of natural products, which could be applied in searching for neuroactive compounds and in elucidating the underlying mechanisms of their neuroprotective activities.

## Figures and Tables

**Table 1:** Chemical compositions of *L. rhinocerus *sclerotial extracts.

Extracts	Total Carbohydrate	Total Protein	Total Phenolic Content
	(mg glucose/g extract)	(mg protein/g extract)	(mg GAE/g extract)
HA	797.86 ± 54.89^a^	113.96 ± 6.97^a^	13.01 ± 0.53^a^
CA	300.22 ± 34.72^bc^	164.46 ± 6.48^b^	16.01 ± 0.21^b^
RT	446.95 ± 13.27^b^	177.02 ± 2.62^b^	16.85 ± 0.26^b^
ME	237.07 ± 24.08^c^	159.31 ± 0.78^b^	20.87 ± 0.75^c^

Results were expressed as mean ± SEM of triplicate measurements (n = 3). Total phenolic content is expressed as mg gallic acid equivalents (GAE) in 1 g extract. Different letters in the same column (a – c) represent significant difference between means (*P* < 0.05).

## Data Availability

The data used to support the findings of this study are included within the article.

## Abbreviations

CA: Cold aqueous extract
DEX: Dexamethasone
HA: Hot aqueous extract
hESC: Human embryonic stem cell
ME: Methanol
MNs: Mature neurons
NSCs: Neural stem cells
pAkt: Phospho-Akt
RT: Room temperature aqueous extract.

## Supplementary Materials

Supplementary Materials
* Figure S1:* Characterization and fluorescence microscopy of the* in vitro *models. (A) NSCs expressing Nestin exhibited green fluorescence. (B) MNs and (C) differentiated SH-SY5Y cells expressing doublecortin (DCX) and *β*3-tubulin (TUJ-1) were stained in red and green, respectively. Nuclei were counter-stained with DAPI (blue). Scale bar: 100 *μ*m.* Figure S2:* Effects of boiled* L. rhinocerus *(A) HA, (B) ME, (C) CA, and (D) RT extracts on the cell viability of NSCs, MNs, and differentiated SH-SY5Y cells after 24 h of treatment. Results are expressed as mean ± SEM (n = 3). Asterisks denote significant differences between the original extract and boiled extract at 1000 *μ*g/ml; *∗∗∗ P *< 0.001.
