# Supplementary material for: Human Embryonic Stem Cell-Derived Neural Lineages as In Vitro Models for Screening the Neuroprotective Properties of Lignosus rhinocerus (Cooke) Ryvarden
Source: Biomed Res Int. 2019 Aug 19;2019:3126376. doi: 10.1155/2019/3126376 (PMC7658738; doi:10.1155/2019/3126376)
Supplement: Supplementary Materials — Figure S1: Characterization and fluorescence microscopy of the in vitro models. (A) NSCs expressing Nestin exhibited green fluorescence. (B) MNs and (C) differentiated SH-SY5Y cells expressing doublecortin (DCX) and β3-tubulin (TUJ-1) were stained in red and green, respectively. Nuclei were counter-stained with DAPI (blue). Scale bar: 100 μm. Figure S2: Effects of boiled L. rhinocerus (A) HA, (B) ME, (C) CA, and (D) RT extracts on the cell viability of NSCs, MNs, and differentiated SH-SY5Y cells after 24 h of treatment. Results are expressed as mean ± SEM (n = 3). Asterisks denote significant differences between the original extract and boiled extract at 1000 μg/ml; ∗∗∗ P < 0.001. [file 3126376.f1.pdf]

Supplementary Figures

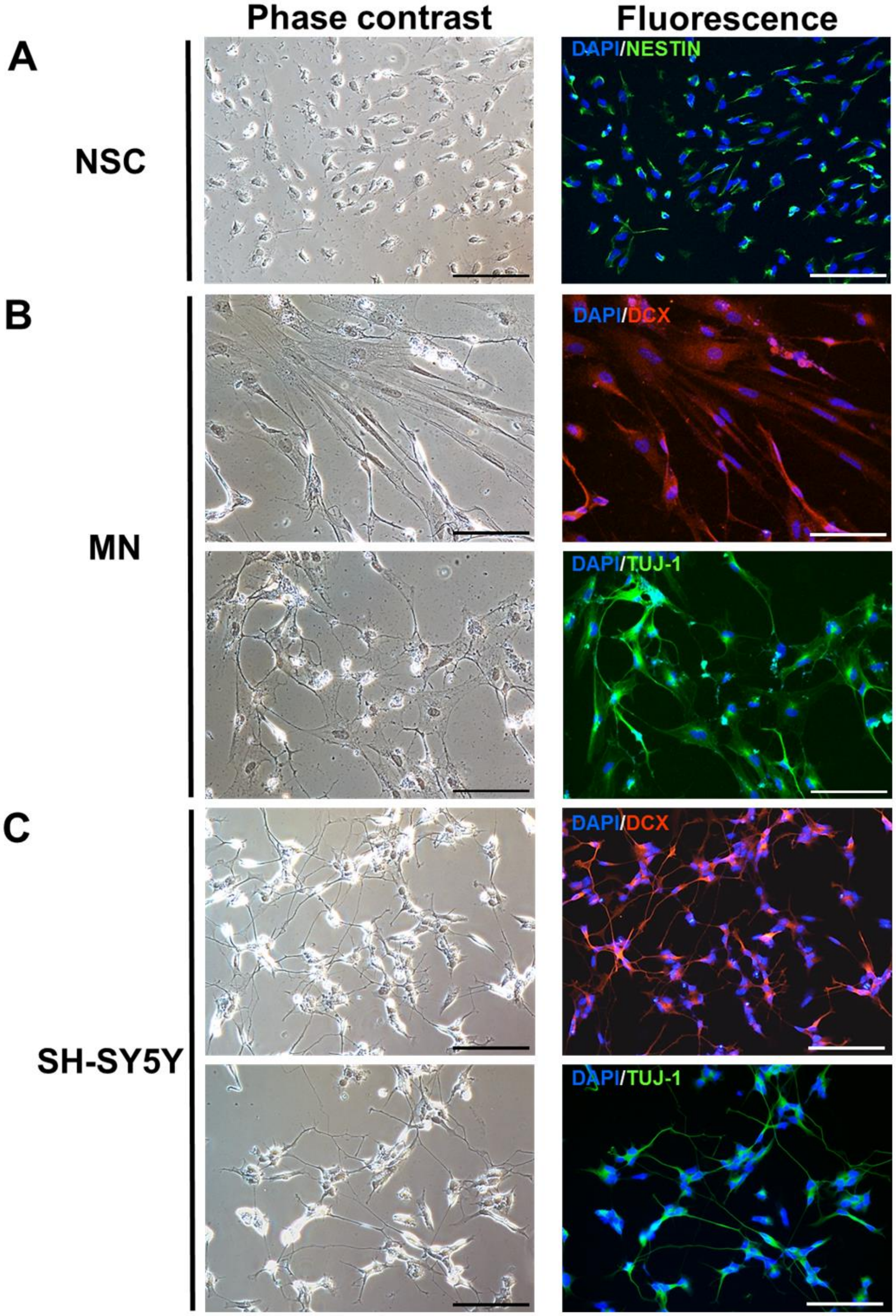

Figure S1: Characterization and fluorescence microscopy of the *in vitro* models. (A) NSCs expressing Nestin exhibited green fluorescence. (B) MNs and (C) differentiated SH-SY5Y cells expressing doublecortin (DCX) and β3-tubulin (TUJ-1) were stained in red and green, respectively. Nuclei were counter-stained with DAPI (blue). Scale bar: 100 μm.

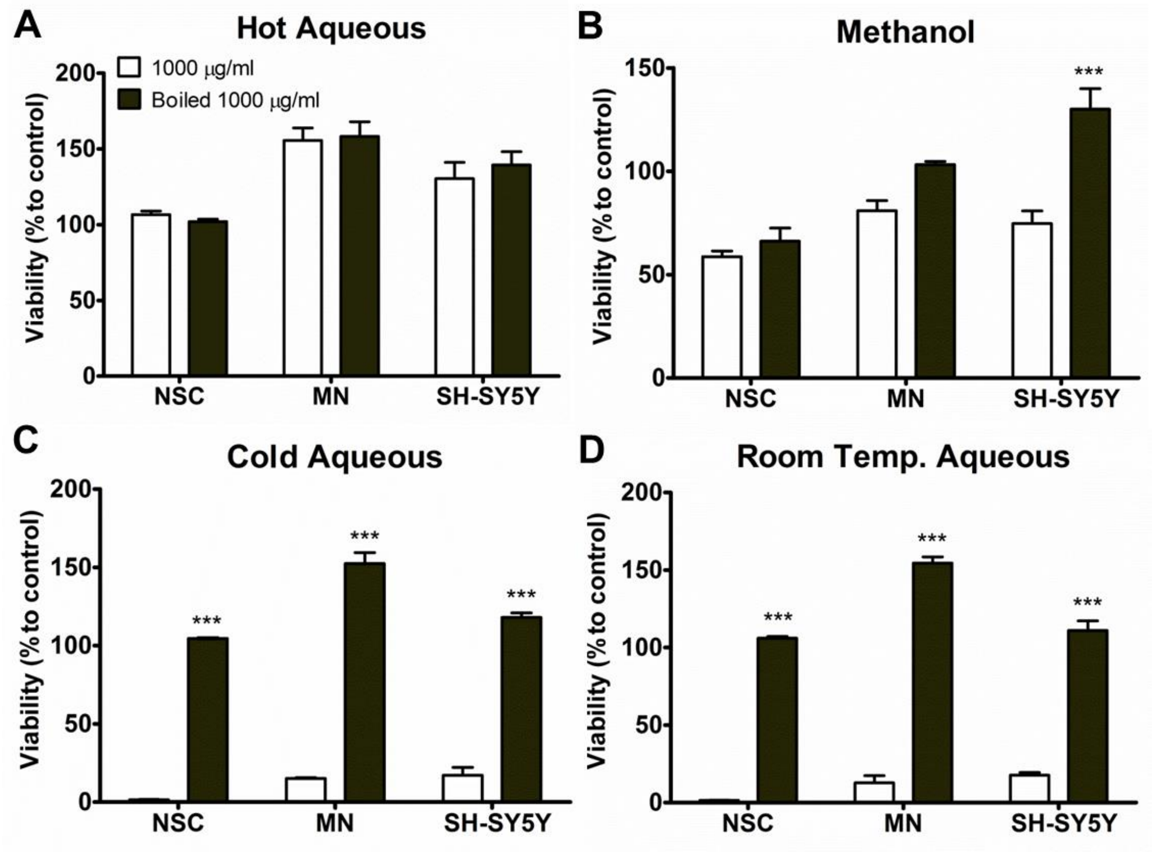

Figure S2: Effects of boiled *L. rhinocerus* (A) HA, (B) ME, (C) CA and (D) RT extracts on the cell viability of NSCs, MNs and differentiated SH-SY5Y cells after 24 h of treatment. Results are expressed as mean ± SEM (n = 3). Asterisks denote significant differences between the original extract and boiled extract at 1000 μg/ml; *** $P < 0.001$.
